# Acquisition cancer stemness, mesenchymal transdifferentiation, and chemoresistance properties by chronic exposure of oral epithelial cells to arecoline

**DOI:** 10.18632/oncotarget.11432

**Published:** 2016-08-20

**Authors:** Tung Yuan Wang, Chih-Yu Peng, Shiuan-Shinn Lee, Ming-Yung Chou, Cheng-Chia Yu, Yu-Chao Chang

**Affiliations:** ^1^ School of Dentistry, Chung Shan Medical University, Taichung, Taiwan; ^2^ Department of Dentistry, Chung Shan Medical University Hospital, Taichung, Taiwan; ^3^ Institute of Oral Sciences, Chung Shan Medical University, Taichung, Taiwan; ^4^ School of Public Health, Chung Shan Medical University, Taichung, Taiwan

**Keywords:** arecoline, cancer stemness, oral squamous cell carcinomas

## Abstract

Oral squamous cell carcinoma (OSCC), one of the most deadliest malignancies in the world, is caused primarily by areca nut chewing in Southeast Asia. The mechanisms by which areca nut participates in OSCC tumorigenesis are not well understood. In this study, we investigated the effects of low dose long-term arecoline (10 μg/mL, 90-days), a major areca nut alkaloid, on enhancement cancer stemness of human oral epithelial (OE) cells. OE cells with chronic arecoline exposure resulted in increased ALDH1 population, CD44 positivity, stemness-related transcription factors (Oct4, Nanog, and Sox2), epithelial-mesenchymal transdifferentiation (EMT) traits, chemoresistance, migration/invasiveness/anchorage independent growth and *in vivo* tumor growth as compared to their untreated controls. Mechanistically, ectopic miR-145 over-expression in chronic arecoline-exposed OE (AOE) cells inhibited the cancer stemness and xenografic. In AOE cells, luciferase reporter assays further revealed that miR-145 directly targets the 3′ UTR regions of Oct4 and Sox2 and overexpression of Sox2/Oct4 effectively reversed miR-145-regulated cancer stemness-associated phenomenas. Additionally, clinical results further revealed that Sox2 and Oct4 expression was inversely correlated with miR-145 in the tissues of areca quid chewing-associated OSCC patients. This study hence attempts to provide novel insight into areca nut-induced oral carcinogenesis and new intervention for the treatment of OSCC patients, especially in areca nut users.

## INTRODUCTION

In spite of the recent advancements in the multidisciplinary treatment for oral squamous cell carcinoma (OSCC), OSCC remains one of the leading causes of cancer-related mortality worldwide [1]. Extensive epidemiologic evidence have demonstrated increased risk for the development of OSCC associated with areca nut chewing in Southeast Asia [2, 3]. Mounting short-term assay studies have demonstrated that arecoline, a major areca nut alkaloid, contributes pathogenesis of OSCC [4, 5]. Nevertheless, the underlying mechanisms by which the long-term arecoline participates in tumorigenesis of OSCC are not well understood.

Recent studies have revealed that cancer stem cells (CSCs) or termed tumor initiating cells (TICs) with tumors could contribute to tumor maintenance, metastasis, radio-resistance and chemo-resistance in a variety of cancers, including OSCC [6–11]. Moreover, the first connection of between CSCs and epithelial-mesenchymal transdifferentiation (EMT), a dynamic process in which cells lose epithelial features and gain mesenchymal properties, is demonstrated in breast cancer stem cells model [12]. Mani et al. found that mammary epithelial cells treated with EMT inducer TGFβ increased cancer stemness marker [12]. Our previous report has shown that S100A4, a mediator of EMT, plays a crucial role the regulation of cancer stemness and tumorigenic properties both *in vitro* and *in vivo* [13]. Overexpression of cancer stemness marker CD133 also increased EMT transformation in OSCC [8]. Therefore, understanding the relationships between areca nut and CSCs/EMT is important to improve further OSCC therapeutics.

In this study, we developed a chronic arecoline-treated oral epithelial cells model for phenotypic and molecular characterization of the arecoline-induced cancer stemness and EMT. The aim of this study was to explore whether long-term chronic arecoline treatment positively correlated with cancer stemness and EMT in OE cells. To the best of our knowledge, we found that long-term arecoline treatment enhanced the *in vitro* and *in vivo* tumorigenicity of OE cells, which could be blocked by miR-145 delivery. This study might open a new avenue for cancer stemness generation by areca nut and to be able to develop innovative treatments for areca nut-associated OSCC patients.

## RESULTS

### Elevation of cancer stemness marker ADLH1 activity and CD44 positivity in long-term arecoline-exposed oral epithelial cells

**Figure 1:**
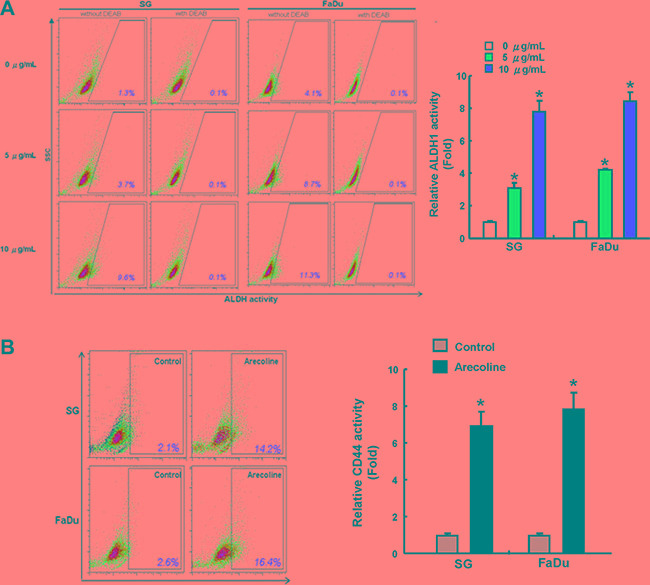
Increase of ALDH1 and CD44 activity in oral epithelial cells with long-term arecoline exposure The expression of ALDH1 activity (**A**) and CD44 positivity (**B**) of control and long-term arecoline exposed oral epithelial cells (SG and FaDu) was determined by flow cytometry analysis. Data shown here are the mean ± SD of three independent experiments. *p < 0.05 vs. Control.

The mechanism by which chronic areca nut treatment progresses to OSCC is poorly described. We hypothesize that one of the mechanisms contributing to the oral carcinogenesis involves cancer stemness enhancement. Two oral epithelial cell lines, SG and FaDu cells, were treated with arecoline up to three months for cancer stemness evaluation compared with their parental cells. Mounting reports suggested that aldehyde dehydrogenase (ALDH) activity [14] and CD44 [7] expression could be the common markers to identify the oral CSCs. Our data reported that long-term arecoline sustained treatment dose-dependently elevated ALDH1 activity of OE cells (Figure 1A). CD44 expression levels were also higher in arecoline-exposed OE cells, but lower in parental OE cells (Figure 1B).

### Chronic arecoline treatment increases sphere-forming capacity and the expression of stemness markers

**Figure 2:**
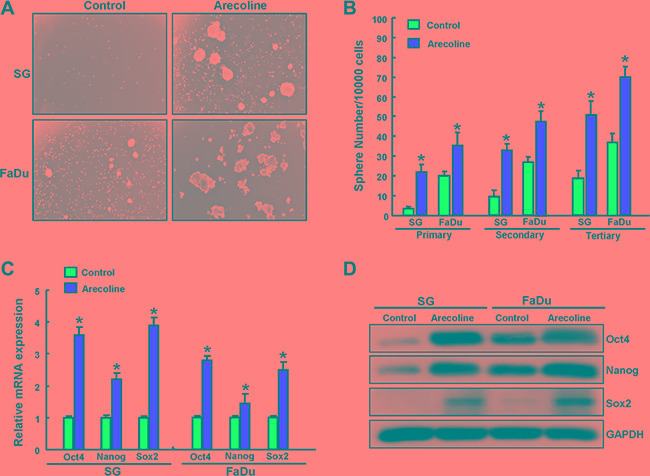
Enhanced self-renewal capacity and pluripotency genes in long-term arecoline-exposed oral epithelial cells (**A**) Representative image of control and long-term arecoline exposed oral epithelial cells (SG and FaDu) culturing in a serum-free medium were subjected to secondary oralspheres formation assays. (**B**) The secondary oralspheres generated from control and long-term arecoline exposed OE cells during three passages were quantified. (**C**) qRT-PCR analysis and (**D**) Western blot analysis of Nanog, Oct4 and Sox2 expression in the control and long-term arecoline exposed OE cell subclones. Data shown here are the mean ± SD of three independent experiments. *p < 0.05 vs. Control.

Oralsphere formation over serial passages of culture is gold standard methodology for evaluating the self-renewal property in CSCs [15]. Accordingly, the spheres body size (Figure 2A), efficiency of secondary sphere/tertiary sphere formation (Figure 2B) efficiency was increased after chronic arecoline exposure. Real-time RT-PCR (Figure 2C) and western blotting analysis (Figure 2D) also showed up-regulation of stemness markers expression (Oct4, Nanog, and Sox2) in arecoline-exposed OE cells compared with their parental cells.

### Chronic arecoline-exposed oral epithelial cells showed chemoresistence to cisplatin and 5-FU

**Figure 3:**
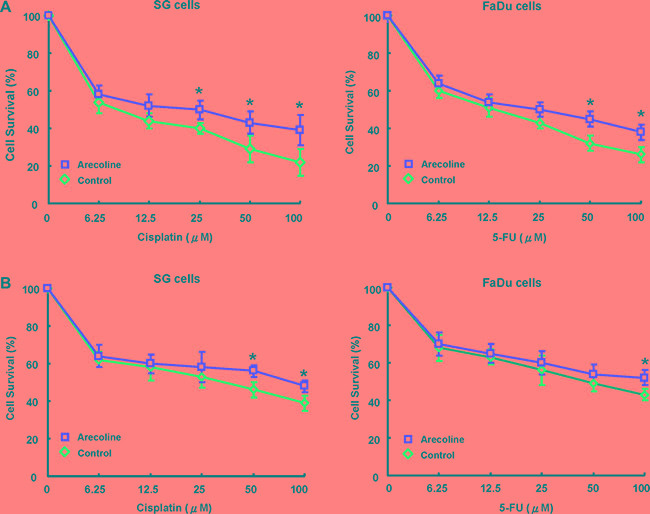
Long-term arecoline exposure increased chemoresistance in oral epithelial cells Control and long-term arecoline exposed oral epithelial cells were subjected to treatment with different concentrations of cisplatin (**A**) and 5-FU (**B**). Cell viability was determined by MTT assay (*p < 0.05).

The observation of arecoline-enhanced the CSCs population and its properties suggested their involvement in modulating the chemoresistance, an important hallmark of CSCs [16]. To explore the expression of arecoline-exposed OE cells and chemoresistance, control and arecoline-exposed OE cells treated with chemotherapeutic agents and subjected to MTT analysis. MTT analysis revealed that OE cells with long-term arecoline exposure significantly increased chemoresistence to cisplatin (Figure 3A) and 5-FU (Figure 3B) compared to the parental OE cells.

### Increased tumorigenicity and EMT properties in oral epithelial cells with long-term arecoline exposure

**Figure 4:**
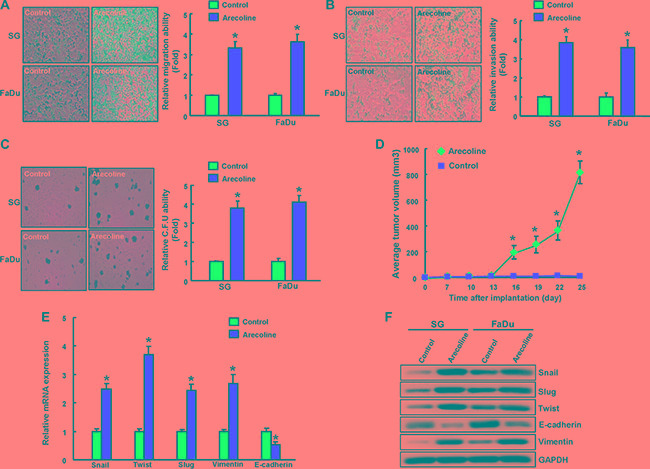
Enhanced *in vitro* and *in vivo* oncogenicity in long-term arecoline-stimulated oral epithelial cells Control and long-term arecoline exposed oral epithelial cells were subjected to migration assay (**A**), matrix invasion assay (**B**), and a soft agar colony formation assay (**C**). (**D**) Xenograft tumor volume in recipients of 1 × 10^4^ control (yellow arrow) and long-term arecoline-treated OE cells (red arrow) were monitored over 25 day experimental period. Expression of EMT-related markers in control and long-term arecoline-treated OE cells was determined by (**E**) qRT-PCR analysis and (**F**) Western blot analysis. The amount of GAPDH protein of different crude cell extracts was referred as loading control for further quantification. Data shown here are the mean ± SD of three independent experiments. *p < 0.05 vs. Control.

To assess the impact of chronic arecoline exposure on tumorigenicity *in vitro* and *in vivo*, we performed migration/invasion assay using transwell system and colony-forming assay. Migration (Figure 4A), invasion (Figure 4B) and colony formation (Figure 4C) were also enhanced in long-term arecoline-exposed OE cells when compared with the corresponding untreated OE cells. Comparing to control OE cells, xenotransplantation of long-term arecoline-treated OE cells induced severe tumor formation in immunocompromised mice at 25 day post-transplantation (Figure 4D). Epithelial-mesenchymal transition (EMT), a de-differentiation program that converts adherent epithelial cells into individual migratory cells, is thought to be a cellular process commonly associated with CSCs [12]. Real-time RT-PCR analysis also demonstrated increased transcripts of mesenchymal markers and reduced epithelial marker in chronic arecoline-exposed OE (AOE) cells (Figure 4E). Consistently, AOE cells also increased protein levels of mesenchymal markers and reduced epithelial marker by western blotting (Figure 4F).

### miR-145 ablated arecoline-induced cancer stemness and *in vivo* tumorigenesis

**Figure 5:**
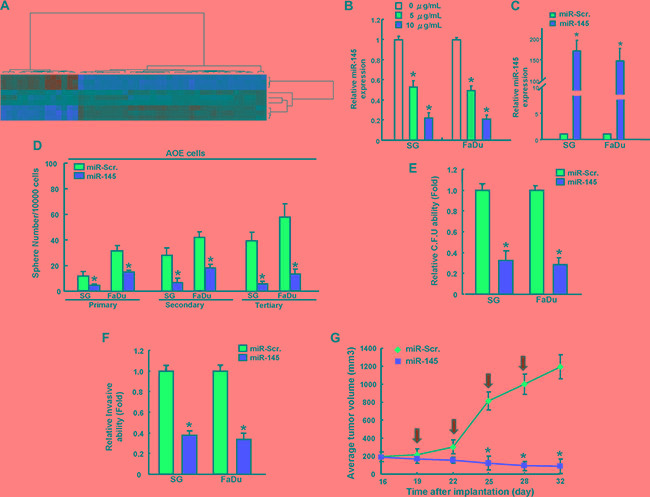
miR-145 effectively reverses long-term arecoline treatment-induced cancer stemness *in vitro* and *in vivo* (**A**) The heat maps of control and long-term arecoline-treated oral epithelial cells were subjected to miRNA microarray and bioinformatics analysis. A significant decrease in miR-145 expression is found in long-term arecoline-treated oral epithelial cells. qRT-PCR analysis showing miR-145 in long-term arecoline exposed OE cells (**B**) and theses cells were transfected with miR-145 overexpression vector (**C**). These miR-145-transfected long-term arecoline exposed OE subclone cells were then assigned for the sphere formation assay (**D**), colony-forming assay (**E**), invasion assay (**F**), and xenografic tumorigenesis in nude mice with IVIS imaging system (**G**). Data shown here are the mean ± SD of three independent experiments. *p < 0.05 vs. Control.

MicroRNAs (miRNAs), a class of small noncoding RNAs regulating the gene expression by binding to the 3′ untranslated region (UTR) of target mRNAs, have been involved in cancer stemness and EMT during carcinogenesis [17]. miRNAs microarray analyses identified miR-145, the known tumor suppressive microRNA, is significant down-regulated in AOE cells (Figure 5A). Consistent with the miRNA microarray results, long-term arecoline exposure dose-dependently led to the marked down-regulation of miR-145 expression by miRNA real-time RT-PCR analysis (Figure 5B). To further investigate the effect of miR-145 on biological properties of AOE cells, we gained-of function miR-145 in AOE cells through lentiviral-mediated transduction (Figure 5C). miRNA real-time RT-PCR analysis confirmed the miR-145 over-expression effect in AOE cells (Figure 5C). The sphere-forming ability among the primary, secondary and tertiary spheres was consistently impaired in AOE cells with miR-145 overexpression (Figure 5D). The formation of soft agar colonies (Figure 5E) and invasiveness ability (Figure 5F) was suppressed in AOE cells after miR-145 over-expression. *In vivo* recipients of xenografts of AOE cells that received delivery of miR-145 lentivirus exhibited attenuated tumor formation (Figure 5G).

### miR-145 directly targets Oct4 and Sox2 and Oct4/Sox2 co-expression mediates miR-145-regulated cancer stemness and EMT

**Figure 6:**
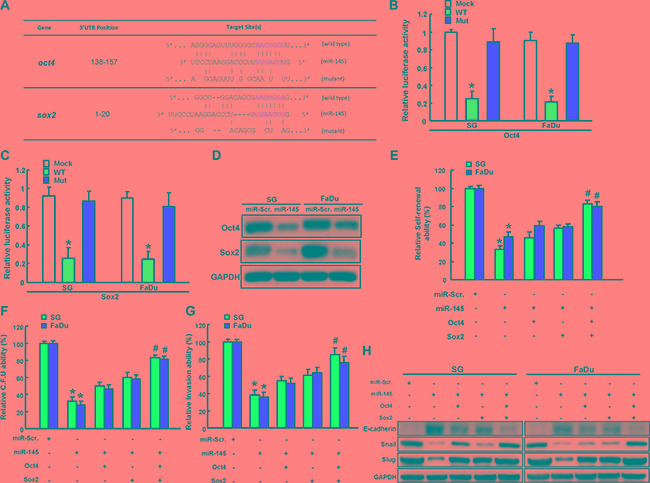
miR-145 regulated by long-term arecoline treatment targets Sox2 and Oct4 (**A**) Schematic presentation of the constructed Sox2 and Oct4 3′UTR reporter plasmids were used in this study. The wild-type and mutated (Mut) Oct4 (**B**) and Sox2 (**C**) reporter plasmids were co-transfected with miR-145 or empty vector. The luciferase activity of each combination was assessed and was presented with wild-type (WT) and mutated (Mut) reporter plasmids. The results of the luciferase assays indicated that only WT reporter activity was inhibited by miR-204. (**D**) The protein expression levels of Sox2 and Oct4 in transfected indicated plasmids were analyzed by western blot. Long-term arecoline-stimulated oral epithelial cells were transfected with miR145, SOX2, and Oct4 individually or concurrently indicated before being subjected to secondary sphere-forming (**E**), colony-forming ability assay (**F**), and invasion assay (**G**). The percentage or fold-change is presented in the chart. (**H**) Cells with indicated vectors transfection were then subjected to western blot analysis for the expression level of EMT markers expression.

Subsequently, we illustrate complementarity between the 3′UTR regions of Oct4 and Sox2 and miR-145. To pinpoint the miR-145 target sequences in the 3’UTRs of Oct4 and Sox2, reporter plasmids which contained either full-length or mutated forms of the 3′UTR region of Oct4 and Sox2 were constructed (Figure 6A). Luciferase reporter assays demonstrated that miR-145 reduced the luciferase activity of reporter plasmids containing wild type Oct4 (Figure 6B) and Sox2 (Figure 6C) 3′UTR. However, when the potential Oct4 and Sox2 targeting site was mutated, miR-145 no longer inhibited the luciferase activity (Figure 6B and Figure 6C). Protein levels of Oct4 and Sox2 were also decreased in the miR-145-overexpressing AOE cells (Figure 6D). The functional involvement of Oct4 and Sox2 in miR-145-mediated cancer stemness and EMT in AOE cells was further clarified through Oc4/Sox2 overexpression. miR-145 suppressed spheres-forming capability in AOE cells, which would be rescued by Oc4/Sox2 over-expression (Figure 6E). Furthermore, Oc4/Sox2 over-expression in miR-145-overexpressing AOE cells partially counteracted clonogenicity (Figure 6F) and invasion phenomenons (Figure 6G). We demonstrated that miR-145 down-regulated a pattern of mesenchymal-like transcription factor (Snail and Slug) and up-regulated epithelial protein (E-cadherin) in AOE cells, were reversed by Oct4/Sox2 overexpression (Figure 6H).

### Negative correlation of miR-145 and Oct4/Sox2 expression in areca nut-associated OSCC patients

**Figure 7:**
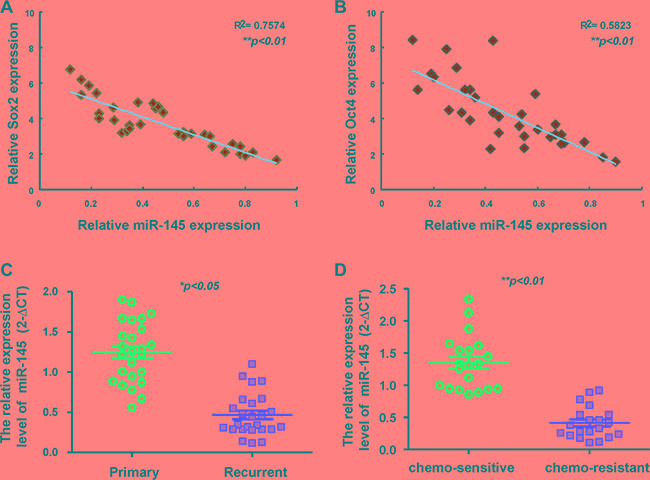
Clinical relevance of miR-145 in OSCC patients An inverse correlation can be seen between miR-145 /Sox2 (**A**) and miR-145 /Oct4 (**B**) in OSCC patients’ tissues. (**C**) OSCC tissue samples from primary lesions (P) and recurrent (R) lesion were subjected to real-time RT-PCR analysis for the expression levels of miR-145. (**D**) OSCC tissue samples from chemo-sensitive and chemo-resistant lesions were subjected to real-time RT-PCR analysis for the expression levels of miR-145. **P < 0.01; paired *t*-test was used in this sample cohort.

To determine significant correlation between miR-145 and Oct4/Sox2, the expression of miR-145 and Oct4/Sox2 among OSCC patients was analyzed by qRT-PCR and Spearman rank correlation tests. qRT-PCR analysis shows a negative correlation between miR-145 expression and Sox2 mRNA expression in OSCC specimen revealed by linear regression analysis (Figure 7A). A significant inverse correlation between miR-145 expression and Oct4 expression was also found using the OSCC tumor samples (Figure 7B). The expression of miR-145 in OSCC tissues was significantly decreased in the recurrent tumor specimens, while miR-145 expression in primary OSCC tissues was higher relative to the recurrent OSCC tissue (Figure 7C). We also found down-regulation of miR-145 in chemo-resistant than in chemo-sensitive OSCC tissues (Figure 7D).

## DISCUSSION

Epidemiological analysis has demonstrated the majority of OSCC arise in Southeast Asia are due to areca nut chewing together with tobacco smoking [18]. Nevertheless, the pathogenetic roles of areca nut in OSCC have not been well addressed. Epithelial to mesenchymal transition (EMT) process has been implicated as major contributor to oncogenic progression, cancer stemness and cancer metastasis [19]. Previous studies have shown that areca nut extract or arecoline could activate several EMT-related molecules, such as vimentin [20], Snail [21], and ZEB1 [4] in oral epithelial or fibroblastic cells. These interesting findings suggested a possible link between areca nut and EMT/stemness during oral carcinogenesis. In the study, we established chronic arecoline-exposed oral epithelial (AOE) cells. Using our model, these cells exhibit higher capability in oralsphere formation, populations of ADLH-positive cell induction, oncogenicity *in vitro* and *in vivo*, and potential for EMT conversion (Figures 1–4). We expect the study is to extend our approaches to insight the influence of arecoline in oncogenic and cancer stemness induction *in vitro* and *in vivo*, may allow us to develop potential targeting therapy against OSCC.

A growing number of recent studies have focused on environmental carcinogens exposure leading modulation of tumor malignancy through cancer stemness conversion. Arsenic [22–24] or nickel [25] exposure induced cell transformation through CSCs accumulation. Nicotine or its derivative 4-methylnitrosamino-1-3-pyridyl-1-butanone (NNK) could facilitate CSCs population generation in OSCC [26], breast cancer, colorectal cancer [27], pancreatic cancer [28]. Chronic ethanol exposure can increase the aggressiveness and CSCs of breast cancer cells via p38γ MAPK/RhoC signalings [29]. The areca nut chewing, tobacco, and together with alcohol consumption are the best known etiological factors for the development of OSCC. Future research would be required to examine whether the synergistic effect of these risk factors are involved in CSCs enhancement during OSCC carcinogenesis.

MicroRNAs (miRNAs) are short, non-coding RNAs that regulate gene expression either by translational inhibition or by degradation of the targeted mRNA [30]. It is also well known that dysregulation of miR-145 has been reported in several human solid tumors [31]. miR-145 has been found to be key role in suppressing tumor for OSCC development. miR-145 inhibits cancer stemness in various cancer types, including lung cancer [32], hepatocarcinoma [33], prostate cancer [34], and glioma [35]. This molecule is also associated with the cell cycle [36], differentiation [37], and apoptosis [38], metastasis [39], and chemoresistance [40]. Here, we confirmed that miR-145 negatively regulates cancer stemness in chronic arecoline-exposed OE (AOE) cells. Additionally, we found that Oct4/Sox2 stemness genes are targets of miR-145 through its binding to the Oct4 and Sox2 3′-UTR (Figure 6A). Co-overexpression of Sox2 and Oct4 rescued the repression effect of miR-145 on cancer stemness (Figure 6E–6G). As the Sox2/Oct4 also modulate the EMT of OSCC [41, 42], the regulation of the Sox2/Oct4 by miR-145 that modulate EMT and cancer stemness needs to be further investigated.

In conclusion, chronic arecoline exposure induces malignant phenotype with the acquisition of cancer stemness/EMT, and oncogenicity *in vitro* and *in vivo*. miR-145 might be partial mechanism of areca nut-induced OSCC. Validation and detail molecular understanding of later genes are ongoing to determine the arecoline associated pathway alterations during the OSCC tumorigenesis. A full understanding of the mechanism underlying arecoline-induced oral cancer may help to design a more effective strategy to treat areca nut-associated OSCC patients and prolong life.

## MATERIALS AND METHODS

### Cell culture and reagents

The Smulow–Glickman (S-G) human gingival epithelial and FaDu OSCC cell lines were used in this study. The cultivation of these cells was following the protocols previously used.

Arecoline was purchased from Sigma Chemical Co. (St. Louis, MO, USA) and was dissolved in ddH_2_O as a stock solution of 100 mM. Just before use, arecoline was further diluted in culture medium to appropriate final concentrations [41].

### Cancer stemness-associated phenotypic analysis

The phenotypes of including oralspheres formation, migration [10], invasion [10], soft agar colony forming assay, ALDEFLUOR assay [9], CD44+ population by flow Cytometry analysis [43] was conducted according to previously used protocols.

### Quantitative real-time PCR (qRT-PCR)

Total RNA is prepared from cells using Trizol reagent according to the manufacturer's protocol (Invitrogen Life Technologies, Carlsbad, CA, USA). qRT–PCRs of mRNAs are reverse-transcribed using the Superscript III first-strand synthesis system for RT–PCR (Invitrogen Life Technologies, Carlsbad, CA, USA). qRT-PCR reactions on resulting cDNAs were performed on an ABI StepOne™ Real-Time PCR Systems (Applied Biosystems).

### Western blot analysis

Western blot analysis was followed previously described protocols [4]. The primary antibodies will be those against Oct4, Nanog, Sox2, Snail, Twist, Slug, E-cadherin, and Vimentin [4].

### Constructs

miR-145 overexpression plasmid constructs were generated according to our previous methods [9]. The Sox2 and Oct4 3′ UTRs were amplified by PCR and were cloned into the pMIR-REPORT vector (Applied Biosystems). Human full-length Oct4 and Sox2 cDNA was cloned into pCDH1-MCS1-EF1-copGFP (System Biosciences, Cat. No: CD511A-1; Mountain View, CA, USA). Lentivirus production was performed as previously [9].

### Xenograft cancer mouse model

All the animal practices in this study has been approved and in accordance with the Institutional Animal Care and Use Committee (IACUC) of Chung Shan Medical University university. Cells from each stable miR-145 -overexpressing or miR-Scramble cells will injected subcutaneously into BALB/c nude mice (6–8 weeks). Tumor volume (TV) will be calculated using the following formula: TV (mm^3^) = (Length × Width ^2^) / 2 [6].

### OSCC tissue subjects

With the permission of the institutional review board of Chun Shan Medical University, resected tissues from OSCC patients, who gave informed consent for the use of their tissue, were harvested at surgery. OSCC tumor samples were subjected to qRT-PCR analysis.

### Statistical analysis

Statistical Package of Social Sciences software (version 13.0) (SPSS, Inc., Chicago, IL) was used for statistical analysis. Student's *t* test was used to determine statistical significance of the differences between experimental groups; *p* values less than 0.05 were considered statistically significant. The level of statistical significance was set at 0.05 for all tests.
